# Supplementary material for: Cloning and functional characterization of porcine AACS revealing the regulative roles for fat deposition in pigs
Source: PeerJ. 2023 Nov 20;11:e16406. doi: 10.7717/peerj.16406 (PMC10666648; doi:10.7717/peerj.16406)
Supplement: Supplemental Information 1 — Reaction systems and reaction procedures for reverse transcription cDNA. [file peerj-11-16406-s001.docx]

**Supplementary method:**

**Reaction systems and reaction procedures for reverse transcription cDNA：**

Incubate the 10 μL reaction mixture (2 μL gDNA buffer, 2 ng RNA, and ddH2O) at 42 ℃ for 3 minutes. Then add 2 μL of 10× Fast RT Buffer, 1 μL of RT Enzyme Mix, 2 μL of FQ-RT Primer Mix, and RNase-Free ddH2O to make a total volume of 20 μL. Incubate at 42 ℃ for 15 minutes, followed by incubation at 95 ℃ for 3 minutes.

**Cloning and sequence analysis of AACS**

All PCR products were gel purified and cloned into the PUC19 vector (included in the SMARTER® RACE 5′/3′ kit). The recombinant plasmid was sequenced using SinoGenoMax, and the full-length porcine AACS mRNA sequence was ligated using DNAMAN8 software (Lynnon Biosoft). The amino acid hydrophobicity was predicted using the Prot Scale in ExPASy (https://web.expasy.org/protscale/). ExPASy online software (https://web.expasy.org/protparam/) was used to predict the theoretical pI, net charged residues, and instability index of AACS.
