# Supplementary material for: Cloning and functional characterization of porcine AACS revealing the regulative roles for fat deposition in pigs
Source: PeerJ. 2023 Nov 20;11:e16406. doi: 10.7717/peerj.16406 (PMC10666648; doi:10.7717/peerj.16406)
Supplement: Table S1 [file peerj-11-16406-s002.docx]

Table S1 Primers for gene cloning

| **Name** | **Primer sequence** | **Length**（bp） | **Tm（℃）** |
| --- | --- | --- | --- |
| 3R-2 | GATTACGCCAAGCTTACAGCTGACTGTCAGGGCCATGGG | 577 | 67 |
| 5R-3 | GATTACGCCAAGCTTAGCCAGCCCAGCTCAGAAGTCCTG | 2211 | 65.1 |
| 5R-4 | GATTACGCCAAGCTTCAGAGGCTGTCCATCACCTCCTCG | 1892 | 65.7 |
| AACS-s-F | AACCCTTGTGTTCCCGTGTA | 1575 | 60 |
| AACS-s-R | AACCTGACCCAACTTAGAGC | 1575 | 60 |
