# Supplementary material for: Cloning and functional characterization of porcine AACS revealing the regulative roles for fat deposition in pigs
Source: PeerJ. 2023 Nov 20;11:e16406. doi: 10.7717/peerj.16406 (PMC10666648; doi:10.7717/peerj.16406)
Supplement: Table S2 [file peerj-11-16406-s003.docx]

Table S2. Specific primers for qPCR

| **Genes name** | **Forward primer sequence** | **Reverse primer sequence** |
| --- | --- | --- |
| *Pig-C/EBPα* | CCAAGCCGAGCAAGAAGC | CAGGGCGAACGGGAAAC |
| *AACS* | AAGAGCATCCGCAACGCCAT | CTTCTTGCCGTTGAGGGTGT |
| *Pig-FASN* | AGCCTAACTCCTCGCTGCAAT | TCCTTGGAACCGTCTGTGTTC |
| *AP2* | GAGCACCATAACCTTAGATGGA | AAATTCTGGTAGCCGTGACA |
| *PPARγ* | AGGACTACCAAAGTGCCATCAAA | GAGGCTTTATCCCCACAGACAC |
| *SREBP-1c* | GAGCACCATAACCTTAGATGGA | AAATTCTGGTAGCCGTGACA |
| *pig-CDK4* | CTTTGACCTGATTGGGCTGC | CAGAGATTCGCTTGTGTGGGT |
| *pig-CyclinB* | TGAGGAAGAACAAGCAGTTAGACC | TCACAAAGGCAAAGTCACCAAT |
| *pig-BAD* | TGAGCAGAGTGAGCAGGAAGAC | TGGGTAAGAGCTGTGGCGA |
| *β-actin-pig* | GGACTTCGAGCAGGAGATGG | AGGAAGGAGGGCTGGAAGAG |
