# Supplementary material for: Cloning and functional characterization of porcine AACS revealing the regulative roles for fat deposition in pigs
Source: PeerJ. 2023 Nov 20;11:e16406. doi: 10.7717/peerj.16406 (PMC10666648; doi:10.7717/peerj.16406)
Supplement: Table S3 [file peerj-11-16406-s004.docx]

Table S2. The primer sequences for AACS gene SNP identification.

| **Name** | **Forward primer sequence** | **Reverse primer sequence** | **Length**（bp） |
| --- | --- | --- | --- |
| AACS-5’-1 | AGGAGAAAGGAGCAGAGCCA | TTGAGCAGGGAACGGAAT | 631 |
| AACS-5’-2 | GAAGCCAAATCAAACCAGAG | GTGCCAAGTTTGCTGAGTTA | 933 |
| AACS-5’-3 | GAGGCAACCACACCAGCAAA | GAAACCACCATCCATCAC | 487 |
