# Supplementary material for: Cloning and functional characterization of porcine AACS revealing the regulative roles for fat deposition in pigs
Source: PeerJ. 2023 Nov 20;11:e16406. doi: 10.7717/peerj.16406 (PMC10666648; doi:10.7717/peerj.16406)
Supplement: Table S4 [file peerj-11-16406-s005.docx]

| **Name** | **Forward primer sequence** | **Reverse primer sequence** | **Amplification region** |
| --- | --- | --- | --- |
| A1 | GGGGCTATCCCTTCTTCTGA | CTGGGGTGCGAGACTACTGG | -2044-+116 |
| A2 | TCACCTCATCTGGTCACATT | CTGGGGTGCGAGACTACTGG | -1493-+116 |
| A3 | GAGGCAACCACACCAGCAAA | GAAACCACCATCCATCAC | -1050-+116 |
| A4 | AATGGCTAAGATGGGATG | AACTCAGCAAACTTGGCACA | -608-+116 |

Table S4. The primer sequences for Dual-luciferase reporter assays.
