# Supplementary figures and images for: Cloning and functional characterization of porcine AACS revealing the regulative roles for fat deposition in pigs
Source: PeerJ. 2023 Nov 20;11:e16406. doi: 10.7717/peerj.16406 (PMC10666648; doi:10.7717/peerj.16406)

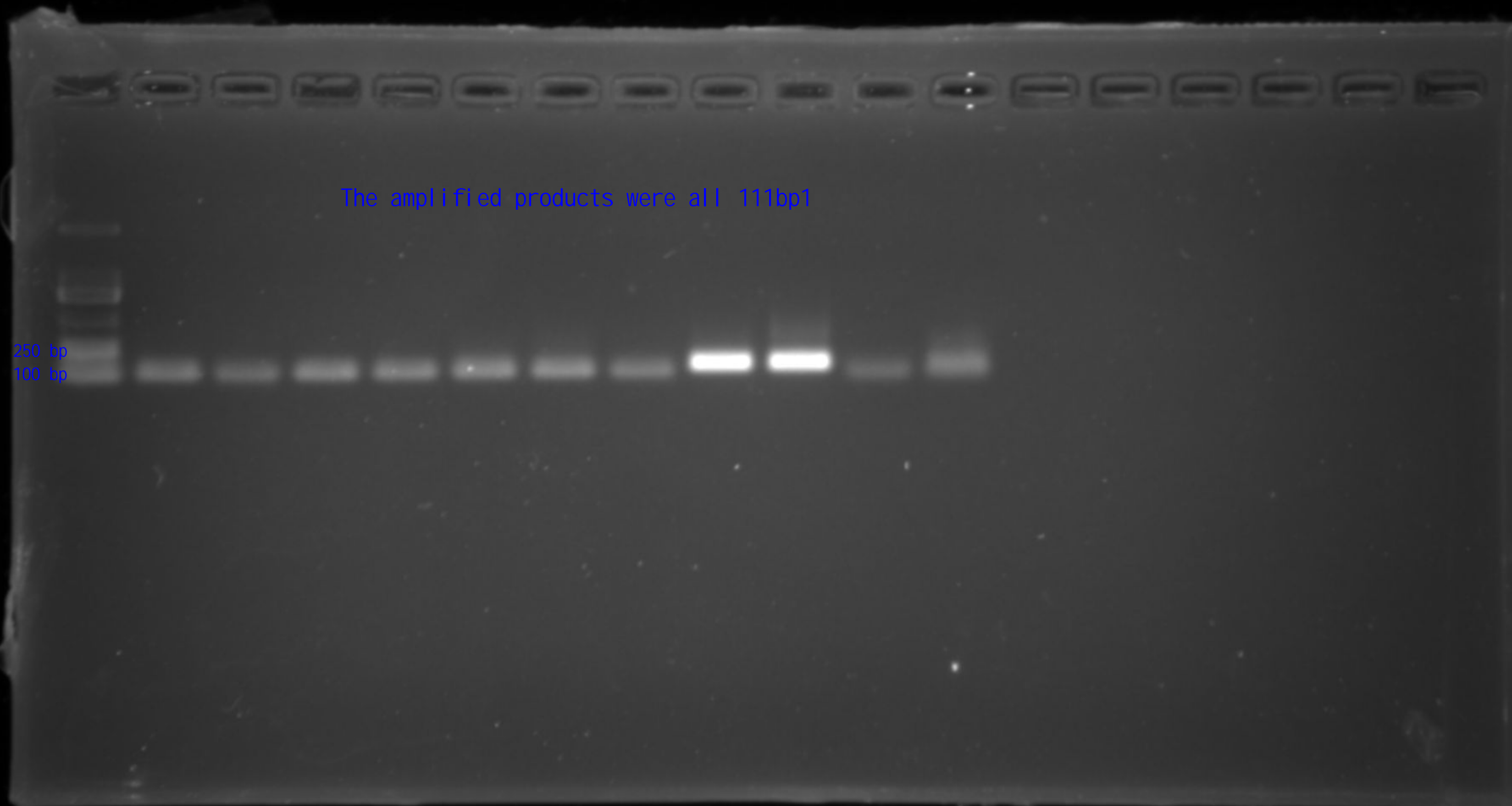

Supplement: Supplemental Information 7 [file peerj-11-16406-s007.zip › uncropped gels/AACS-SqRT-PCR (Figure 2uncropped gels).pdf]

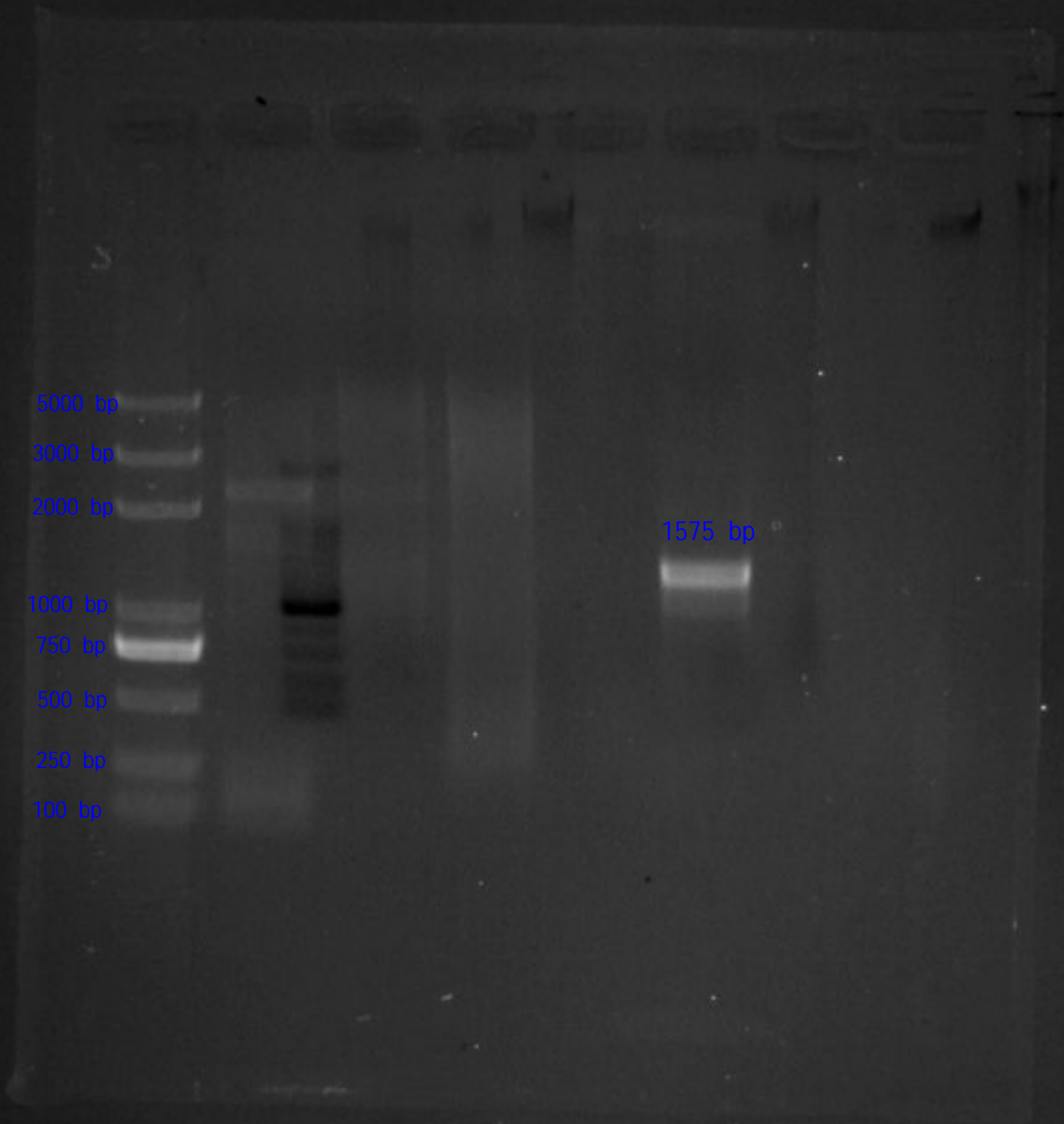

Supplement: Supplemental Information 7 [file peerj-11-16406-s007.zip › uncropped gels/RACE-(Figure 1uncropped gels).pdf]

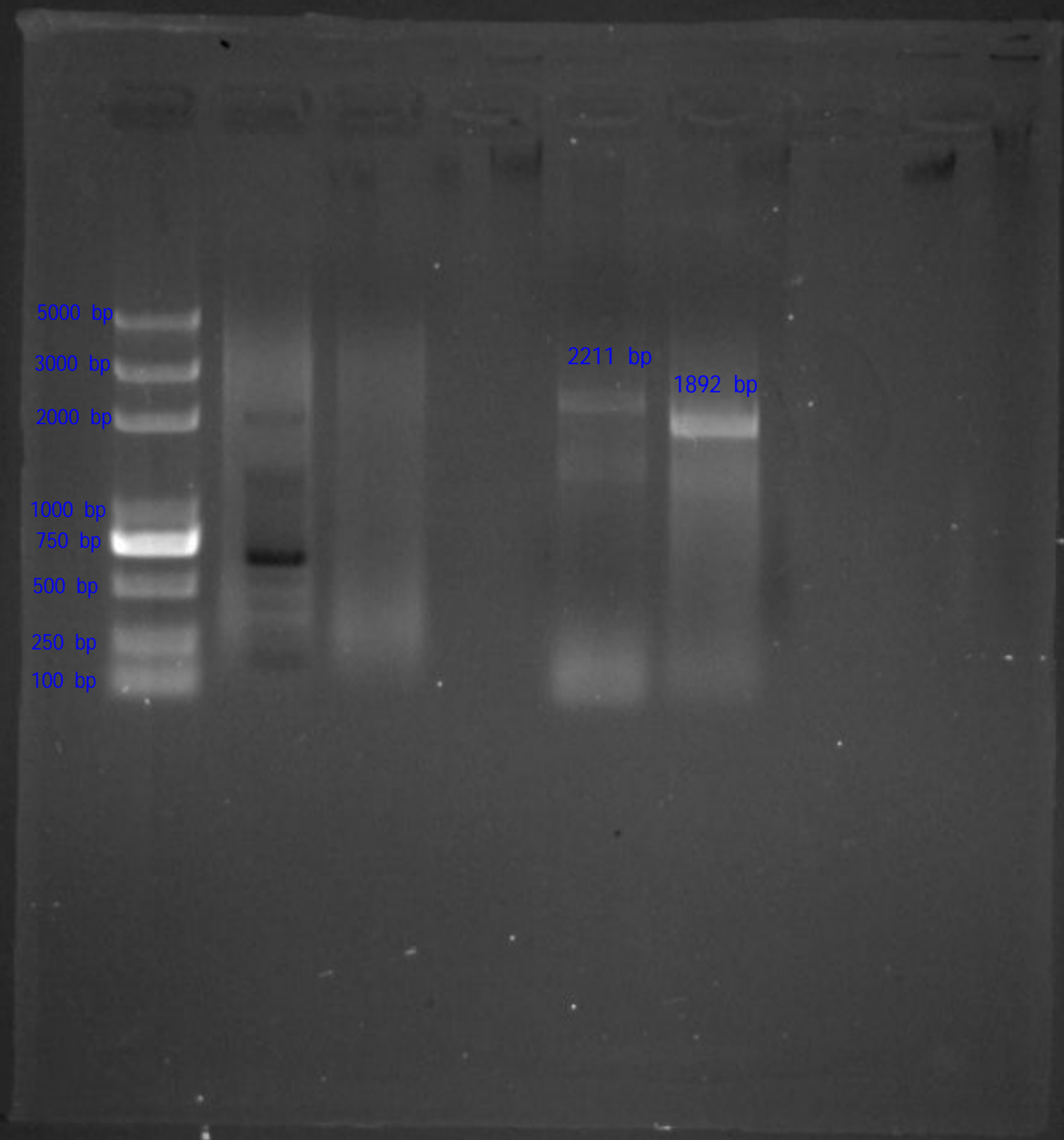

Supplement: Supplemental Information 7 [file peerj-11-16406-s007.zip › uncropped gels/race-3-5(Figure 1uncropped gels).pdf]

The amplified products were all 111bp

750 bp  
500 bp  
250 bp  
100 bp

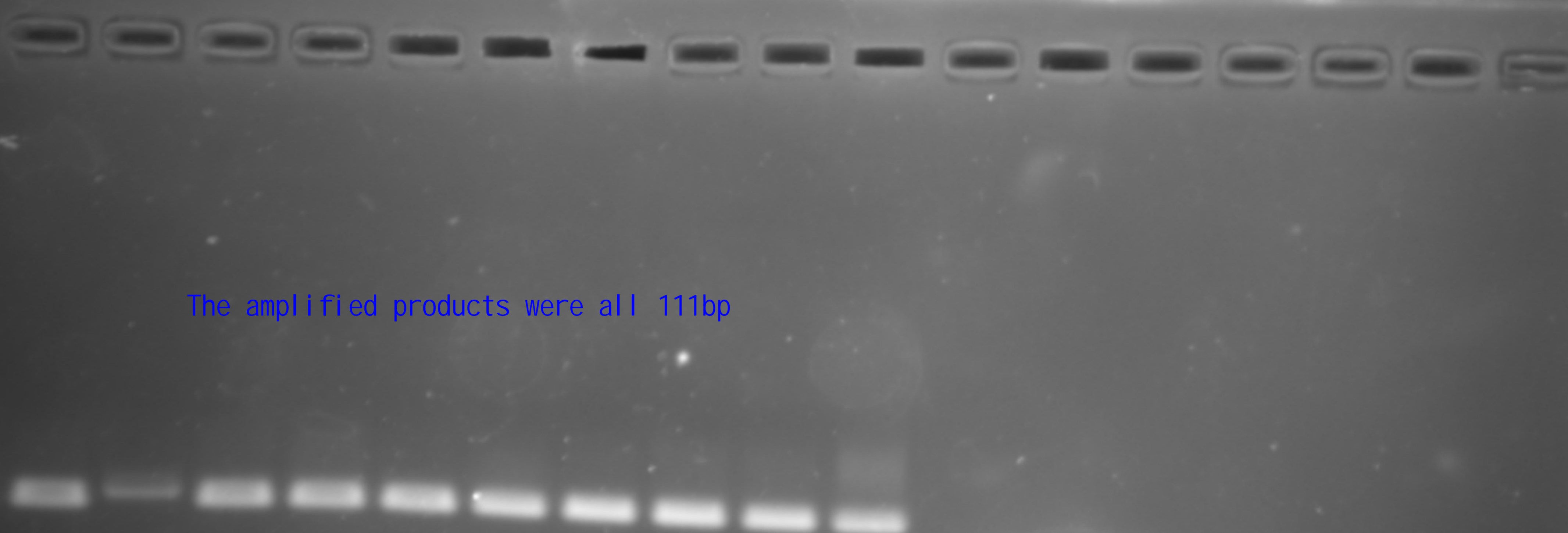

Supplement: Supplemental Information 7 [file peerj-11-16406-s007.zip › uncropped gels/SqRT-PCR-actin(Figure 2uncropped gels).pdf]
